# Melanin Inhibitory Effect of *Tuber himalayense* Isolated in Incheon, Korea

**DOI:** 10.4014/jmb.2311.11021

**Published:** 2024-01-30

**Authors:** Byeong Min Choi, Minkyeong Kim, Hyehyun Hong, Tae-Jin Park, Changmu Kim, Jin-Soo Park, Won-Jae Chi, Seung-Young Kim

**Affiliations:** 1 Department of Pharmaceutical Engineering and Biotechnology, Sunmoon University, Chungnam 31460, Republic of Korea; 2 Biodiversity Research Department Species Diversity Research Division, National Institute of Biological Resources, Incheon 22689, Korea; 3 Natural Product Informatics Research Center, Korea Institute of Science and Technology, Gangneung 25451, Republic of Korea

**Keywords:** α-MSH, B16F10 melanoma cells, MAPK, Melanogenesis, MITF, *Tuber himalayense*

## Abstract

There has been a growing interest in skin beauty and antimelanogenic products. Melanogenesis is the process of melanin synthesis whereby melanocytes are activated by UV light or hormone stimulation to produce melanin. Melanogenesis is mediated by several enzymes, such as tyrosinase (TYR), microphthalmia-associated transcription factor (MITF), tyrosinase-related protein-1 (TRP-1), and TRP-2. In this study, we investigated the effect of *Tuber himalayense* extract on melanin synthesis in α-melanocyte-stimulating hormone (α-MSH)-treated B16F10 melanoma cells. We confirmed that *T. himalayense* extract was not toxic to α-MSH-treated B16F10 melanoma cells and exhibited a significant inhibitory effect on melanin synthesis at concentrations of 25, 50, and 100 μg/ml. Additionally, the *T. himalayense* extract inhibited melanin, TRP-1, TRP-2, tyrosinase, and MITF, which are enzymes involved in melanin synthesis, in a concentration-dependent manner. Furthermore, *T. himalayense* extract inhibited the mitogen-activated protein kinase (MAPK) pathways, such as extracellular signal-regulated kinase-1/2 (ERK), c-Jun N-terminal kinase (JNK), and p38. Therefore, we hypothesized that various components of *T. himalayense* extract affect multiple factors involved in melanogenesis in B16F10 cells. Our results indicate that *T. himalayense* extract could potentially be used as a new material for preparing whitening cosmetics.

## Introduction

Melanin is a phenolic biopolymeric substance widely distributed in nature that inhibits skin aging or sun keratosis due to external factors, such as UV light, and has the potential to protect the skin [1]. However, the abnormal accumulation of melanin not only reduces aesthetic features due to human spots, freckles, and pigmentation, but also promotes cell death by melanin precursors, accelerates skin aging, and causes skin cancer due to toxicity [2, 3]. Melanogenesis is stimulated by UV exposure, stress, and hormones such as α-melanocyte-stimulating hormone (MSH). Melanocyte activation by these stimuli promotes the synthesis of microphthalmia-associated transcription factor (MITF), tyrosinase (TYR), tyrosinase-related protein-1 (TRP-1), and TRP-2 through stress-responsive mitogen-activated protein kinase (MAPK) signaling pathways, such as extracellular signal-regulated kinase-1/2 (ERK), c-Jun N-terminal kinase (JNK), and p38, ultimately causing pigmentation [4-7]. Among these, tyrosinase is an enzyme that acts in the initial rate-determining phase of melanogenesis and catalyzes the hydrolysis of tyrosine to L-3,4-dihydroxyphenylalanine (L-DOPA) and the oxidation of L-DOPA to dopachrome [8]. Two different forms of melanin, black-brown eumelanin and reddish-brown pheomelanin, are also produced by tyrosinase; TRP-1 and TRP-2 are known to be involved in eumelanin synthesis [9]. Recently, as skin features, such as freckles, have increased due to melanin pigmentation caused by strong UV rays, the use of functional cosmetics effective for skin whitening has increased. However, the use of existing whitening functional materials, including hydroquinone, kojic acid, and arbutin, are associated with a number of safety and economic limitations; therefore, research on new whitening materials is necessary. To date, various attempts have been made to inhibit melanogenesis, such as the inhibition of tyrosinase activity, degradation of melanocytes, and reduction and decomposition of melanin, and research on natural products with lower toxicities and fewer side effects is being actively conducted [10-15].

*Tuber* is a genus in the fungal family *Tuber*aceae and is widely known as truffle. Truffle is currently used as a food ingredient due to its reported antioxidant, anti-cancer, and anti-inflammatory activities [16, 17]. In Korea, the genus *Tuber* was only recently discovered, with *T. aestivum* subsp. *uncinatum* being identified in Gyeryongsan National Park in 1995; recently, *T. huidongense* was identified in Pohang in 2020, and *T. koreanum* GB20004 and *T. himalayense* were isolated in Gyeongju and Damyang in 2021 [18, 19]. Many studies on the genus *Tuber* are in progress; however, research on the species native to Korea is inadequate. Among them, *T. himalayense* NIBRFG0000505337, isolated in Incheon, has been reported to have anti-inflammatory activity in previous studies [20]; however, no research has been conducted on its anti-melanogenic activity. Therefore, this study sought to investigate the effects of *T. himalayense* NIBRFG0000505337 on the expression of tyrosinase, TRP-1, TRP-2, and MITF in α-MSH-treated B16F10 melanoma cells. Ultimately, we sought to investigate the potential of *T. himalayense* NIBRFG0000505337 as a whitening agent by assessing its impact on tyrosinase, TRP-1, TRP-2 and MITF expression in α-MSH-treated B16F10 melanoma cells and elucidating the underlying mechanism.

## Materials and Methods

### Chemicals and Reagents

B16F10 melanoma cells were purchased from the American Type Cell Culture Collection (Manassas, VA, USA). Dulbecco’s modified Eagle medium (DMEM), fetal bovine serum (FBS), and penicillin/streptomycin were purchased from Welgene (Republic of Korea). Protease inhibitor cocktail (PI), 3-(4,5-dimethylthiazol-2-yl)-2,5-diphenyltetrazolium bromide (MTT), α-MSH, L-DOPA, and dimethyl sulfoxide (DMSO) were purchased from Sigma-Aldrich (USA). Antibodies against TRP-1, TRP-2, tyrosinase, and MITF were purchased from Santa Cruz Biotechnology (USA). Phospho (p)-p44/42 MAPK (Erk1/2)(Thr202/Tyr204), p-stress-activated protein kinase (SAPK)/JNK, p-p38 MAPK, p-protein kinase B (Akt), p-glycogen synthase kinase-3β (GSK-3β; Ser9)(5B3), p44/42 MAPK (Erk1/2), SAPK/JNK, p38 MAPK, Akt, GSK-3β (27C10), anti-mouse immunoglobulin G (IgG) horseradish peroxidase (HRP), and anti-rabbit IgG HRP antibodies were purchased from Cell Signaling Technology (USA). bicinchoninic acid (BCA) kit was purchased from Pierce Chemial (USA). Radioimmunoprecipitation assay (RIPA) buffer, and enhanced chemiluminescence (ECL) kits were purchased from Bio-Rad (USA).

### *T. himalayense* NIBR0000505337 Extraction

*T. himalayense* NIBR0000505337 was collected on September 27, 2019, from the oak artificial growth zone at the National Institute of Biological Resources in Incheon. Hot water extraction was performed at 121°C for 15 min by adding 1 L of water to 1 g of air dried *T. himalayense*, and ethanol extraction was performed at 25°C for 48 h by adding 1 L of 70% ethanol to 1 g of air dried *T. himalayense* NIBR0000505337. Each extract was filtered through a paper filter (Adventec, Japan), concentrated under reduced pressure, freeze-dried at -110°C, and powdered. The dried product was obtained by concentrating the EA layer in the separated solvent layer by adding ethyl acetate (EA) and was used in all subsequent experiments.

### LC-MS/MS Analysis of *T. himalayense* NIBR0000505337 Extract

Liquid chromatography-tandem mass spectrometry (LC-MS/MS) analysis was performed using a Ultra High Performance Liquid Chromatography (UHPLC) system (Vanquish Flex UHPLC System, Thermo Fisher Scientific, USA) connected to a controller, pump, degasser, autosampler, column oven, and photodiode array detector (ExionLC PDA Detector, Sciex, USA) coupled to a mass spectrometer (Q Exactive UHMR Hybrid Quadrupole-Orbitrap Mass Spectrometer, Thermo Fisher Scientific). The analytical column used was a 100 × 2.1 mm, ACQUITY UPLC BEH C18 column (Waters, USA). Solvent A consisted of acetonitrile, and solvent B consisted of water; each solvent contained 0.1% formic acid. Chromatography was performed at a flow rate of 0.3 ml/min. A linear gradient was programmed for 15 min as follows: 0–10 min, 10% to 100% A; 10–12.5 min, 100% A; 12.5–12.6 min, 100% to 10% A; 12.6–15.0 min, 10% A. The injection volume was 5 μl. Full MS spectra were acquired under positive ionization conditions from 100 to 1,500 m/z at 70,000 FWHM resolution, with MS/MS fragmentation data obtained in the data-dependent scan mode using 30 V collision energies at 17,500 FWHM resolution.

Using the Global Natural Product Social Molecular Networking (GNPS) vendor conversion tool and the file transfer protocol client WinSCP, the LC-MS data of *T. himalayense* NIBR0000505337 extracts were converted to a GNPS-compatible format (.mzXML). On the GNPS analytical platform, under filtration, molecular networks were created by excluding all MS/MS fragment ions that were within 17 Da of the precursor m/z. The MS/MS fragment ion tolerance and precursor ion mass tolerance were set at 2.0 and 0.5 Da, respectively [21].

### Cell Culture

The cells were cultured in DMEM, containing 10% heat-inactivated FBS and 1% penicillin/streptomycin, in a 37°C, 5% CO_2_ humidified incubator. The cells were subcultured every 3 days.

### Measurement of Cell Viability

Cell viability was measured using the MTT assay. Cells were dispensed into 24-well plates at a density of 1 × 10^4^ cells/well and incubated in 37°C, 5% CO_2_ incubator. Approximately 24 h later, cells were treated with α-MSH (200 nM) and various concentrations of the *T. himalayense* NIBR0000505337 hot water extract (THWE) and ethanol extract (THEE) (25, 50, and 100 μg/ml) for 72 h. Thereafter, the MTT solution (1 mg/ml) was added and after 4 h, the medium was removed. The formazan crystals were dissolved in DMSO, and the absorbance was measured at 570 nm using an enzyme-linked immunosorbent assay (ELISA) microplate reader (Thermo Multiskan Go 1510 Sky Microplate Xenon UV/VIS Spectrophotometer Nanodrop, Thermo Fisher Scientific).

### Measurement of Melanin Content

The cells were dispensed into 6-well plates at a density of 4 × 10^4^ cells/well and incubated in in 37°C, 5% CO_2_ incubator for 24 h. Thereafter, cells were treated with α-MSH (200 nM) and various concentrations of the THWE and THEE (25, 50, and 100 μg/ml). After incubation, cells were centrifuged at 800 ×*g* for 3 min. After removing the supernatant, the cells were lysed using RIPA buffer containing 1% PI. Proteins were extracted by vortexing six times at 10-min intervals and then centrifuging at 13,000 ×*g* for 30 min. Thereafter, cells were harvested and dissolved using 500 μl of 1 N NaOH containing 10% DMSO for 1 h at 90°C. The melanin content was measured at an absorbance of 405 nm using an ELISA microplate reader.

### Measurement of Tyrosinase Activity

B16F10 melanoma cells were incubated in a humidified incubator at 37°C and 5% CO_2_, and then dispensed into 6-well plates at a concentration of 4 × 10^4^ cells/well. Approximately 24 h later, the cells were treated with α-MSH (200 nM) and various concentrations of THWE and THEE (25, 50, and 100 μg/ml) for 72 h. After incubation, cells were centrifuged at 800 ×*g* for 3 min. The cells were lysed using RIPA buffer, containing 1% PI, after removing the supernatant. Proteins were extracted by vortexing 6 times at 10-min intervals and then centrifuging at 13,000 ×*g* for 30 min. After lysis, centrifugation was performed for another 30 min (4°C, 13,000 ×*g*) to obtain the supernatant, and the protein content in the supernatant was quantified using a BCA protein assay kit. Thereafter, 80 μl of L-DOPA (2 mg/ml) was added to 20 μl of the quantified protein. The absorbance was measured at a wavelength of 490 nm after 2 h of incubation.

### Western Blotting

B16F10 melanoma cells were pre-incubated in a humidified incubator at 37°C and 5% CO_2_ and dispensed into 6-well plates at a concentration of 4 × 10^4^ cells/well. Subsequently, the cells were treated with α-MSH (200 nM) and THWE (25, 50, and 100 μg/ml) for 48 h or 4 h. Cells were centrifuged at 800 ×*g* for 3 min after incubation. After removing the supernatant, the cells were lysed using RIPA buffer containing 1 mM sodium orthovanadate, 1 mM phenylmethylsulfonyl fluoride, and 1% PI. After lysis, centrifugation was performed for 30 min (4°C, 13,000 ×*g*) to obtain the supernatant, and the protein content in the supernatant was quantified using a BCA protein assay kit. The quantified protein (20 μg) was then subjected to polyacrylamide gel electrophoresis on a 10% sodium dodecyl sulfate gel. After transferring the electrophoresed protein to a polyvinylidene difluoride membrane (Millipore, USA), the membrane was placed in 5% skim milk dissolved in 1× Tris-buffered saline containing 0.1% Tween 20 (TBST) at 25°C for 2 h. Subsequently, the membrane was washed three times at 10-min intervals using 1× TBST, and the primary antibody reaction was conducted at 4°C for 18 h. After the primary antibody reaction, the membrane was washed three times at 10-min intervals using 1× TBST, and the secondary antibody reaction was conducted at 25°C for 2 h. After the secondary antibody reaction, the membrane was washed three times at 10-min intervals using 1× TBST and allowed to react with the reagents of the ECL kit. Proteins were detected using an imaging densitometer (Model GS-700 Imaging Densitometer, Bio-Rad). The expression of the detected proteins was quantified using the ImageJ software (National Institutes of Health, USA) and graphed.

### Statistical Analyses

All experiments were repeated thrice, and the results were expressed as mean ± standard deviation. The data obtained were statistically processed using Student’s *t*-test. *p < 0.05, **p < 0.01, ***p < 0.001.

## Results

### LC-MS/MS Analysis of *T. himalayense* NIBR0000505337

**Fig. 1:**
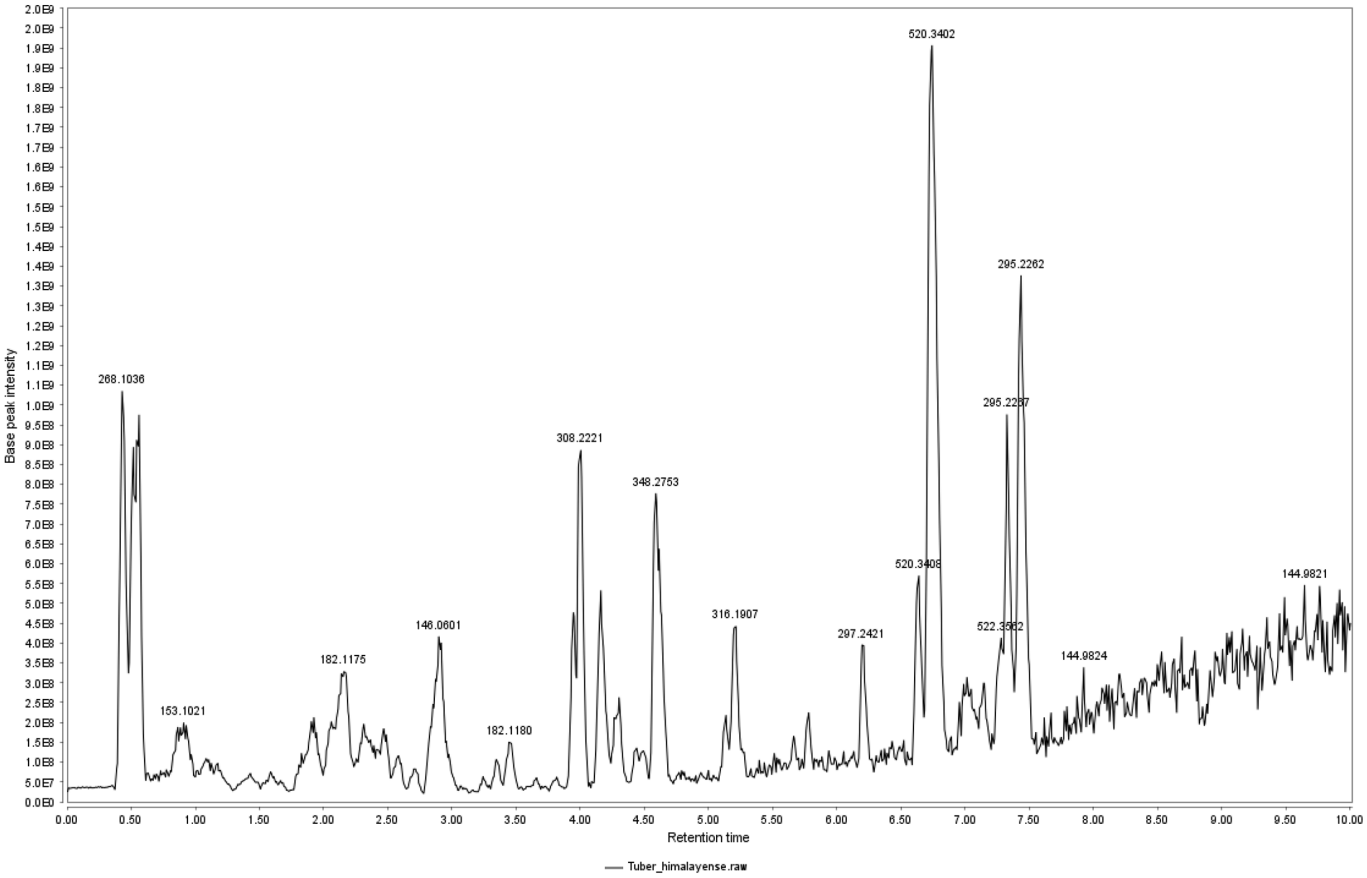
Liquid chromatography-tandem mass spectrometry (LC-MS/MS) analysis of *Tuber himalayense*. Compounds with various m/z values present in the *T. himalayense* extract are displayed at the peaks.

**Fig. 2:**
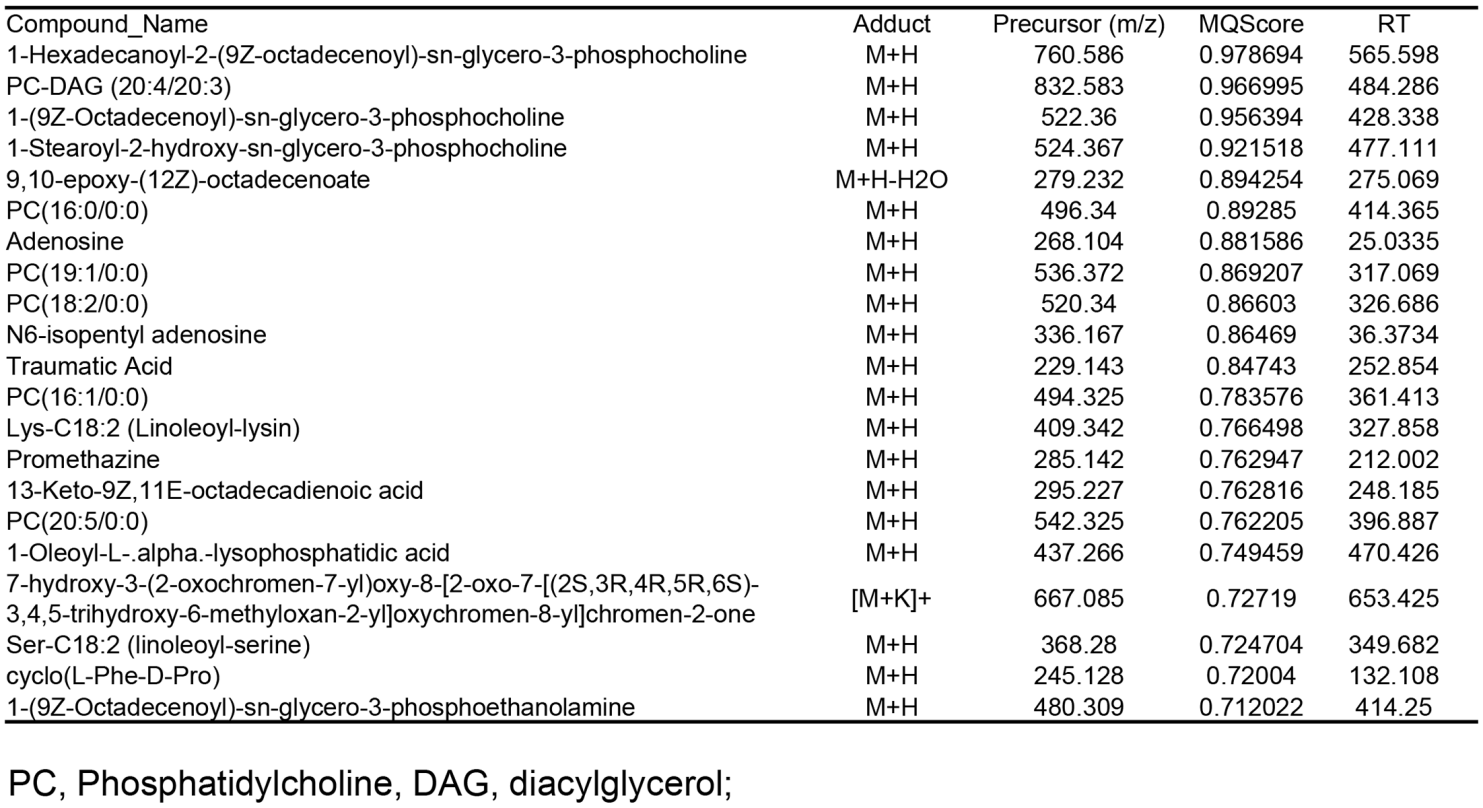
Ingredients of *T. himalayense* extract analyzed using MS/MS fragmentation similarity. A list of the compounds that are likely to exist in the *T. himalayense* extract with a modified cosine score (MQ Score) of 0.7 or higher are listed.

LC-MS/MS analysis of the active ingredients present in *T. himalayense* NIBR0000505337 revealed compounds with peaks of various m/z values (Fig. 1). Using GNPS analysis, which is a web-based mass spectrometry ecosystem that explores highly relevant compounds using MS/MS fragmentation similarity, compounds likely to exist in the extract were identified (Fig. 2). The modified cosine score (MQ Score) was 0.7 or higher, and a total of 22 compounds were thought to exist in the *T. himalayense* NIBR0000505337 extract. Most of the compounds were found to be choline-based, which are commonly present in mushrooms. Furthermore, compounds such as traumatic acid and promethazine reportedly show excellent antioxidant, anti-inflammatory, and skin regeneration abilities. Therefore, further research should be conducted to confirm the presence of these active ingredients in *T. himalayense*.

### Cell Viability of B16F10 Melanoma Cells

**Fig. 3:**
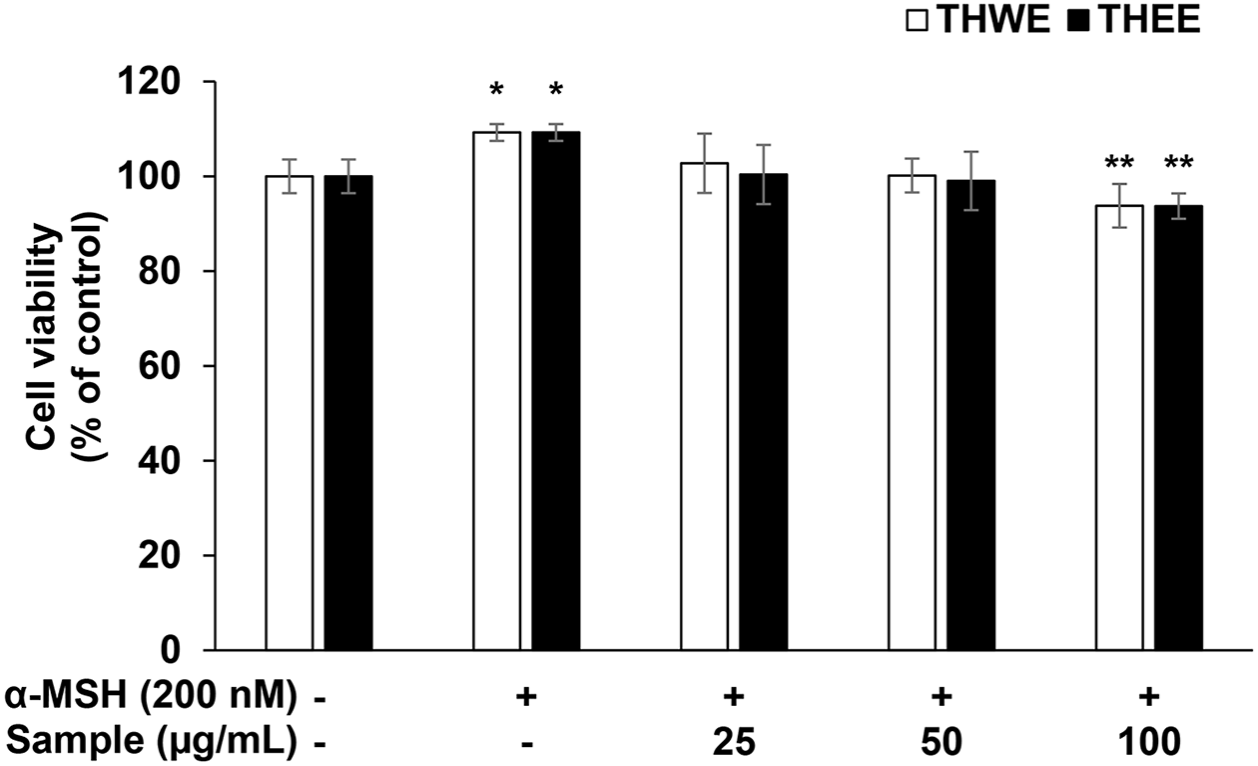
Effect of *T. himalayense* hot water extract (THWE) and *T. himalayense* ethanol extract (THEE) on cell viability of α-melanocyte-stimulating hormone (α-MSH)-treated B16F10 melanoma cells. The cytotoxicity of cells treated with α-MSH (200 nM) in the presence of THWE and THEE (25, 50, and 100 μg/ml) was examined using the MTT assay. Results are expressed as percentages compared with the corresponding values obtained for the control. *p < 0.05; **p < 0.01.

The MTT assay measures cell viability and is based on the ability of mitochondria to reduce MTT tetrazolium, a yellow water-soluble substrate, to water-insoluble MTT formazan with a blue color using dehydrogenase [22]. In this experiment, the MTT assay was performed after simultaneously treating B16F10 melanoma cells with α-MSH (200 nM) and THWE and THEE (25, 50, and 100 μg/ml) to examine the effect of the *T. himalayense* NIBR0000505337 extracts on B16F10 melanoma cell survival. Experiments indicated that THWE and THEE resulted in cell survival rates of >80% at all concentrations (Fig. 3). This suggests that neither sample exhibited toxicity to cells at the measured concentration. Melanin and tyrosinase production inhibition activity was evaluated to confirm the whitening effects of the *T. himalayense* NIBR0000505337 extract.

### Effect of *T. himalayense* NIBR0000505337 on Melanin Production

**Fig. 4:**
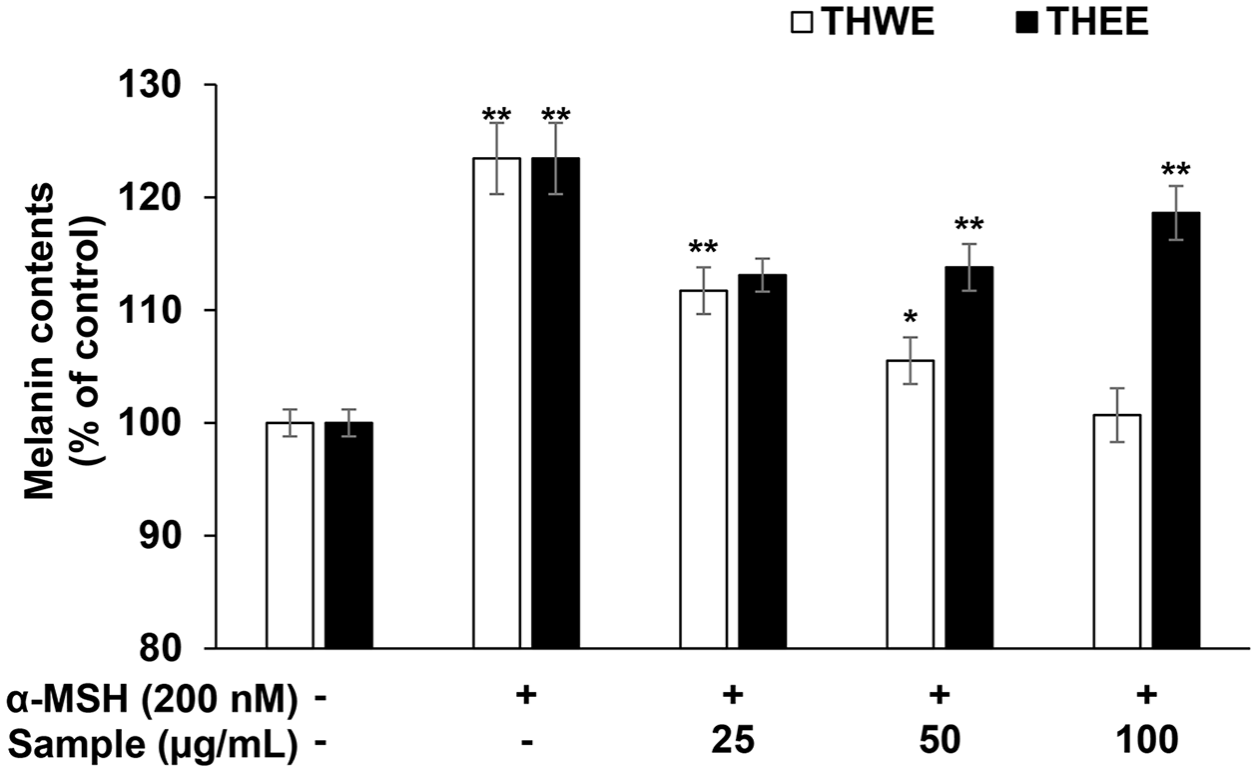
Effect of *T. himalayense* on melanin synthesis of B16F10 melanoma cells. The production of melanin was assayed in the cell pellets of cells stimulated with α-MSH (200 nM) for 72 h in the presence of THWE and THEE (25, 50, and 100 μg/ml). Results are expressed as percentages compared with the corresponding values obtained for the control. *p < 0.05; **p < 0.01.

In this experiment, B16F10 melanoma cells were simultaneously treated with α-MSH (200 nM) and *T. himalayense* extracts (25, 50, and 100 μg/ml), and subsequently cultured to investigate the inhibitory effects of THWE and THEE on melanin production. Thereafter, the cells were lysed to measure the amount of melanin accumulated in the cells. Our results revealed that THWE exhibited melanin inhibitory activity in a concentration-dependent manner. Moreover, the inhibitory activity of THWE was similar to that of the α-MSH untreated group at a concentration of 100 μg/ml of THWE. In contrast, THEE did not exhibit significant inhibitory activity; therefore, THWE was considered to be more effective in suppressing melanin production than THEE (Fig. 4).

### Effect of *T. himalayense* on Tyrosinase Activity in B16F10 Melanoma Cells

**Fig. 5:**
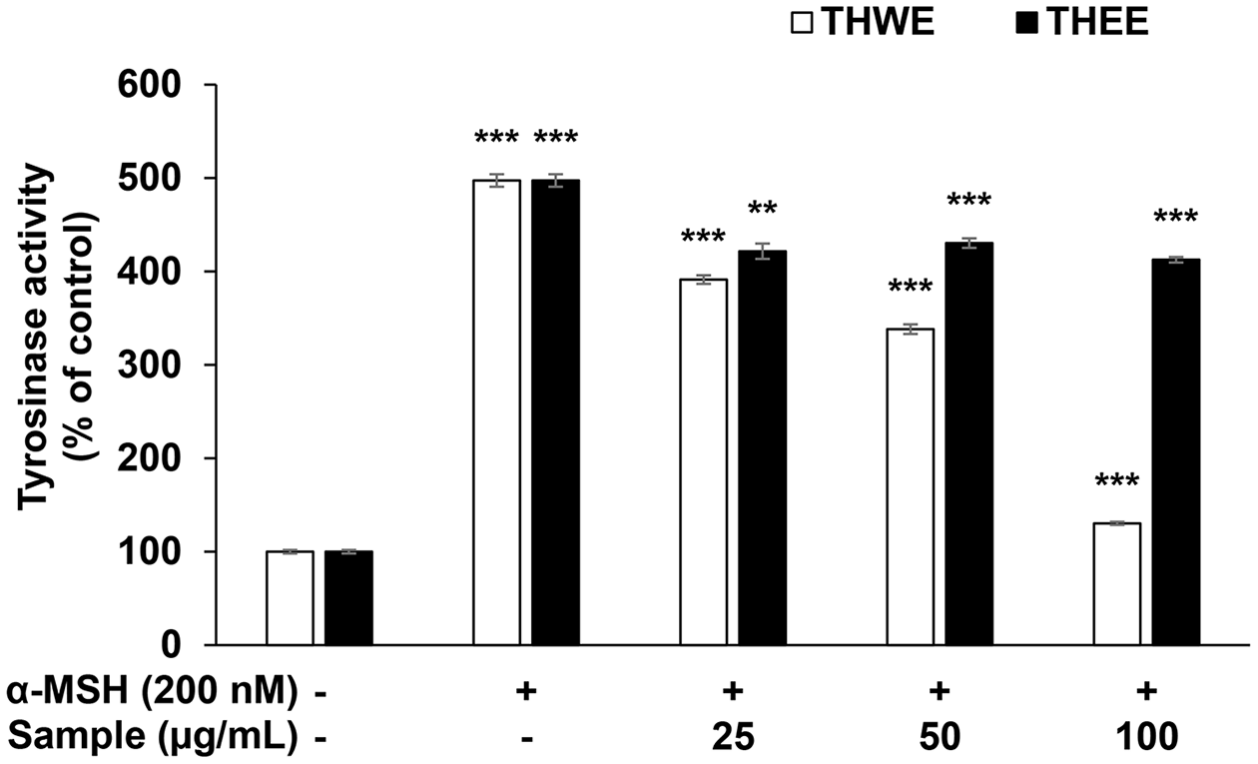
Effect of *T. himalayense* extracts on the tyrosinase activity of B16F10 melanoma cells. The cells were treated with α-MSH (200 nM) for 72 h in the presence of THWE and THEE (25, 50, and 100 μg/ml). The effects of THWE and THEE on tyrosinase activity was investigated by measuring the absorbance at 490 nm. Results are expressed as percentages compared with the corresponding values obtained for the control. *p < 0.05; **p < 0.01; ***p < 0.001.

A crucial enzyme involved in melanogenesis is tyrosinase, which oxidizes tyrosine to L-DOPA-quinone and contributes to melanin synthesis; therefore, the inhibition of tyrosinase production would directly reduce melanin synthesis [23-25]. In this experiment, B16F10 melanoma cells were treated with α-MSH (200 nM) and *T. himalayense* extracts (25, 50, and 100 μg/ml) and subsequently cultured to investigate the effect of THWE and THEE on tyrosinase activity. The amount of tyrosinase produced was measured by extracting proteins from the cells. Our results revealed that the production of tyrosinase decreased in the THWE treatment group in a concentration-dependent manner, whereas no tyrosinase inhibitory activity was observed in the THEE treatment group. In particular, when the cells were treated with THWE at a concentration of 100 μg/ml, we observed tyrosinase inhibitory activity similar to that observed in the α-MSH untreated group (Fig. 5). Based on the melanin and tyrosinase inhibitory activity, THWE was suggested to have significant whitening activity, whereas THEE did not; therefore, all further experiments were conducted using THWE samples.

### Effect of *T. himalayense* NIBR0000505337 on the Protein Expression of TRP-1, TRP-2, Tyrosinase, and MITF

**Fig. 6:**
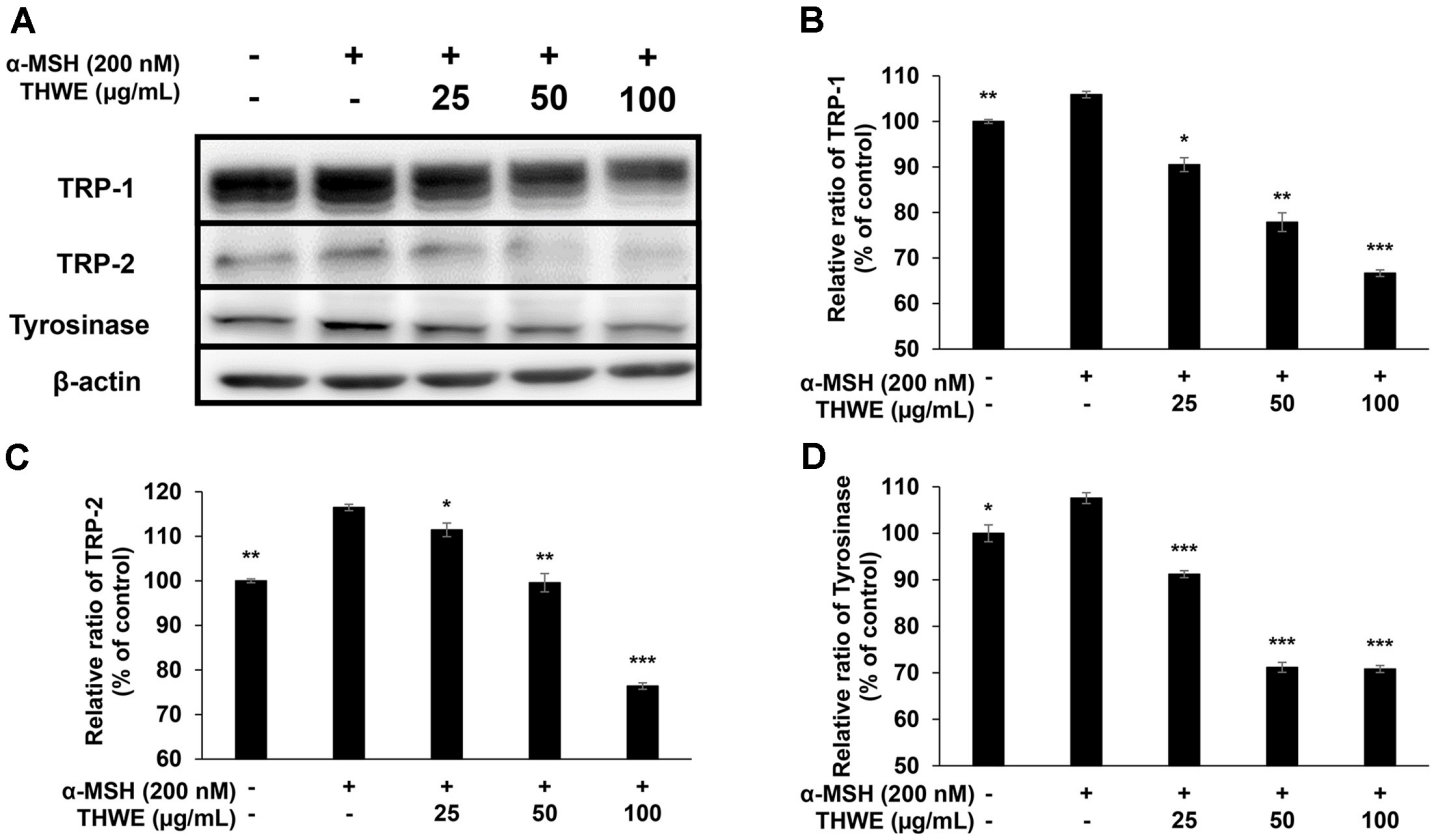
Western blot analysis of the protein levels of tyrosinase-related protein-1 (TRP-1), TRP-2, and tyrosinase in α-MSH-treated B16F10 melanoma cells. Cells were treated with α-MSH (200 nM) and various concentrations of THWE (25, 50, and 100 μg/ml) for 48 h. (**A**) The protein band detection result, (**B**) TRP-1, (**C**) TRP-2, and (**D**) tyrosinase. Data represent the mean ± standard deviation (SD) of values from three separate experiments. *p < 0.05; **p < 0.01; ***p < 0.001.

**Fig. 7:**
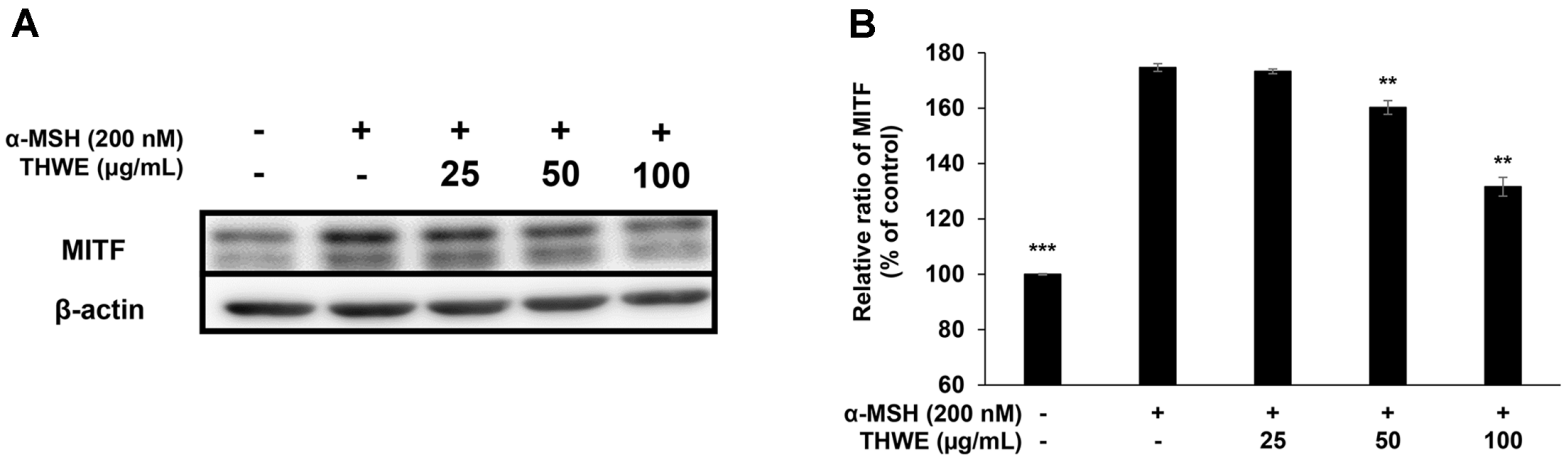
Western blot analysis of the protein levels of microphtalmia-associated transcription factor (MITF) in α-MSH-treated B16F10 melanoma cells. Cells were treated with α-MSH (200 nM) and various concentrations of THWE (25, 50, and 100 μg/ml) for 48 h. (**A**) The protein band detection result, and (**B**) MITF. Data represent the mean ± standard deviation (SD) of values from three separate experiments. *p < 0.05; **p < 0.01; ***p < 0.001.

Tyrosinase catalyzes the oxidation of L-tyrosine to L-DOPA and then to L-DOPA-quinone during melanin synthesis. TRP-1, TRP-2, and MITF also play fundamental roles in melanin synthesis. In this experiment, western blotting was performed to confirm the effect of THWE on the expression of TRP-1, TRP-2, tyrosinase, and MITF. Our results revealed that THWE effectively inhibited the expression of TRP-1, TRP-2, tyrosinase, and MITF (Figs. 6 and 7). This suggests that THWE can be used as a whitening functional raw material that inhibits proteins directly involved in melanin synthesis.

### Effect of *T. himalayense* on MAPK Phosphorylation

Tyrosinase, TRP-1, and TRP-2 are directly involved in melanin production and are regulated by MITF, which is stimulated by various pathways, including the phosphorylation of MAPK, such as ERK, JNK, and p38 [26, 27]. In this experiment, the effect of *T. himalayense* on MAPK phosphorylation was investigated using western blotting. Our results confirmed that *T. himalayense* effectively inhibited the phosphorylation of ERK, JNK, and p38, which function in the MAPK pathway. In particular, the expression of p-p38 was lower than that of the α-MSH untreated group at a THWE concentration of 100 μg/ml. These results confirmed that THWE inhibits the expression of MITF by inhibiting the phosphorylation of ERK, JNK, and p-38, which belong to the MAPK pathway, consequently inhibiting the production of melanin (Fig. 7A-7C).

### Effect of *T. himalayense* on Akt and GSK-3β Phosphorylation

**Fig. 8:**
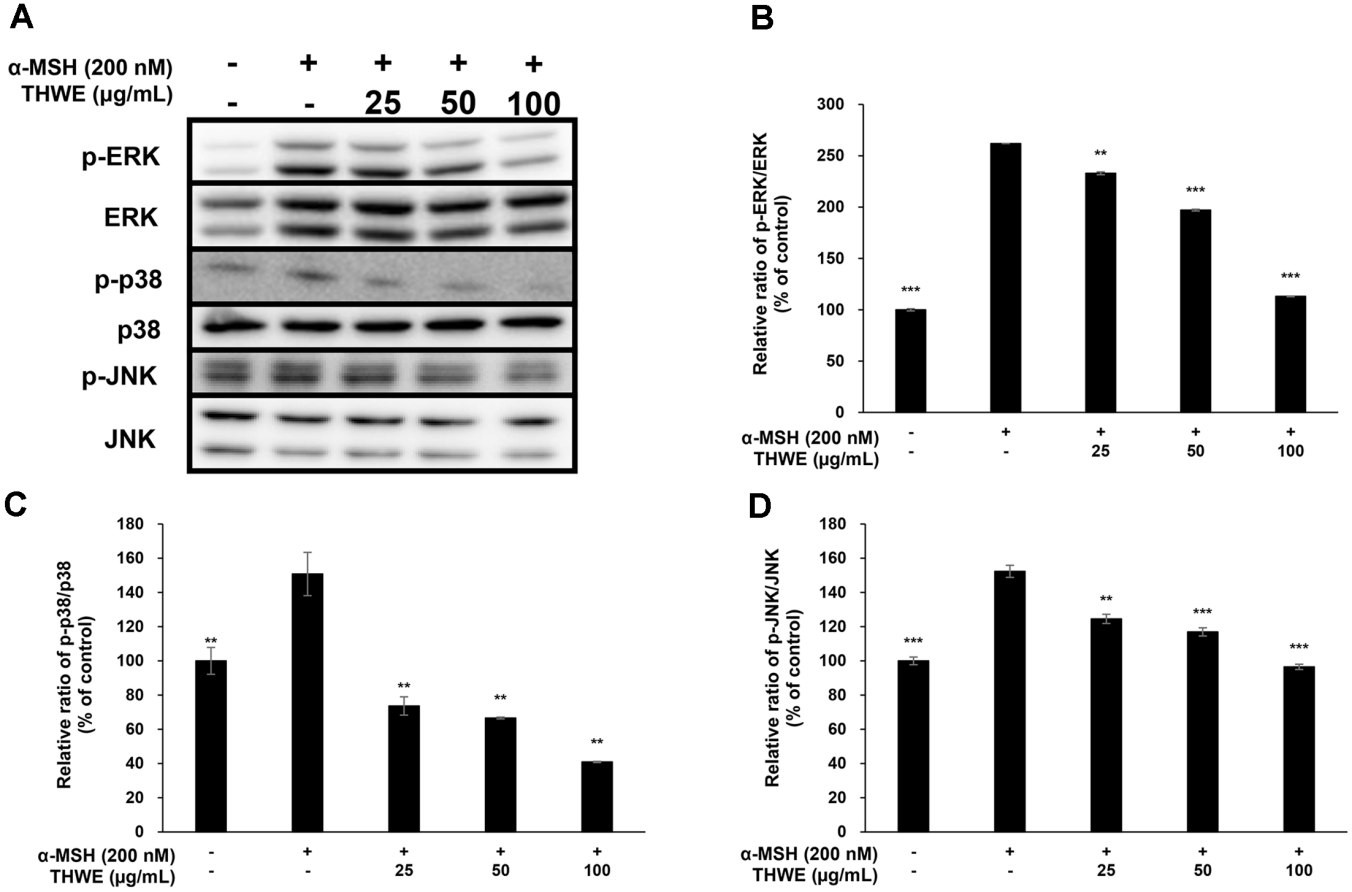
Effect of *T. himalayense* on the phosphorylation of extracellular signal-regulated kinase-1/2 (ERK), c-Jun N-terminal kinase (JNK), and p38. Cells were treated with α-MSH (200 nM) and various concentrations of THWE (25, 50, and 100 μg/ml) for 4 h. (**A**) The proteion band detection results, (**B**) Phospho (p)-ERK/ERK, (**C**) pp38/ p38, and (**D**) p-JNK/JNK. Results are expressed as percentages compared with the corresponding values obtained for the control. Data represent the mean ± SD of values from three separate experiments. *p < 0.05; **p < 0.01; ***p < 0.001.

The expression of MITF, which is directly involved in melanin production, is largely affected by three factors and controlled mainly by the phosphorylation of MAPK, Akt, and cAMP response element-binding protein [28-30]. In this experiment, we explored the effect of *T. himalayense* on the phosphorylation of Akt through western blotting, and the experiment confirmed that *T. himalayense* effectively inhibited the phosphorylation of Akt as well as GSK-3β, a sub-mechanism of Akt (Fig. 8A and 8B). This suggests that the whitening activity of THWE does not occur through a single pathway but through at least two signaling pathways. Based on these results, the potential of THWE as a whitening agent was demonstrated.[Fig F9]

## Discussion

Melanin is an important compound that protects the skin from external stimuli; however, excessive melanin synthesis can lead to pigmentation, freckles, and skin cancer. Melanogenesis is induced by external stimuli, such as UV rays and stress, as well as hormones, such as α-MSH, in the base layer of the skin epidermis. Melanocyte activation by stimuli, such as UV light, promotes the synthesis of melanogenesis-related factors, such as MITF, tyrosinase, TRP-1, and TRP-2, via the phosphorylation of Akt and GSK-3β through the MAPK signaling pathway, including ERK, JNK, and p38 [31-33].

As people's aesthetic needs increase, the use of functional cosmetics, such as anti-wrinkle and whitening agents, has increased. However, due to various limitations associated with synthetic products, there has been an increasing demand for natural cosmetics with fewer adverse effects. Recently, various natural cosmetics have been developed, and as consumer interest in veganism has increased, many researchers are developing cosmetics using plant rather than animal materials [34-36].

*T. himalayense* NIBR0000505337 was first discovered in Korea in 2021. We previously studied the morphological characteristics and anti-inflammatory activity of *T. himalayense* NIBR0000505337 identified in previous Korean studies. However, no research has been conducted on the skin-whitening activity of *T. himalayense* NIBR0000505337. Therefore, in this study, we conducted experiments to verify the skin-whitening activity of *T. himalayense* NIBR0000505337, which was discovered in Korea. Our results revealed that the water extract of *T. himalayense* NIBR0000505337 not only effectively inhibited the expression of tyrosinase, TRP-1, TRP-2, and MITF, but also did not exhibit any signs of toxicity in B16F10 melanoma cells at 25, 50, and 100 μg/ml. Additionally, THWE was confirmed to effectively inhibit the phosphorylation of MAPKs, such as ERK, JNK and p38, as well as the phosphorylation of Akt and GSK-3β, which induce MITF expression. These results suggest that THWE inhibits the synthesis of melanin by inhibiting the expression of MITF, a factor that regulates TRP-1, TRP-2, and tyrosinase. This in turn inhibits the phosphorylation of MAPKs, such as ERK, JNK and p38, and inhibits the phosphorylation of Akt and GSK-3β.

We conducted LC-MS/MS analysis to identify the active ingredients in *T. himalayense* NIBR0000505337, which confirmed that various components, including adenosine, promethazine, and traumatic acid, exist in *T. himalayense* NIBR0000505337, among which, adenosine inhibits melanin synthesis in B16F10 melanoma cells and zebrafish [37]. Further research on the active compounds in *T. himalayense* NIBR0000505337 should thus be conducted. Overall, our results indicated the potential of *T. himalayense* NIBR0000505337 as a raw material for skin whitening in cosmetics.

## Figures and Tables

**Fig. 9 F9:**
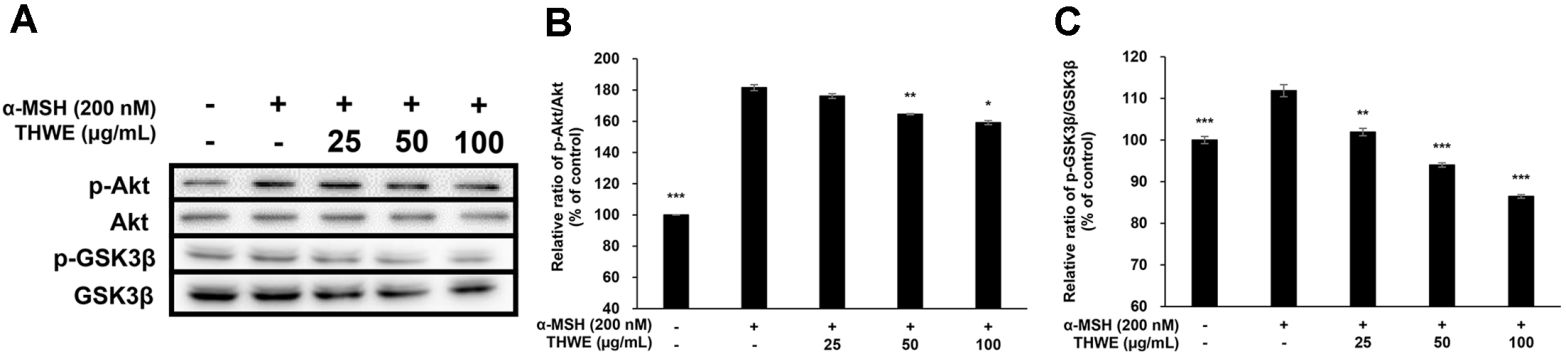
Effect of *T. himalayense* on the phosphorylation of Akt and GSK-3β. Cells were treated with α-MSH (200 nM) and various concentrations of THWE (25, 50, and 100 μg/ml) for 4 h. (**A**) The protein band detection results, (**B**) p- Akt/Akt, and (**C**) p-GSK-3β/GSK-3β. Results are expressed as percentages compared with the corresponding values obtained for the control. Data represent the mean ± SD of values from three separate experiments. *p < 0.05; **p < 0.01; ***p < 0.001.

## References

[1] Solano F, Briganti S, Picardo M, Ghanem G (2006). Hypopigmenting agents: an updated review on biological, chemical and clinical aspects. Pigment Cell Res..

[2] Yoon NY, Eom TK, Kim MM, Kim SK (2009). Inhibitory effect of phlorotannins isolated from *Ecklonia cava* on mushroom tyrosinase activity and melanin formation in mouse B16F10 melanoma cells. J. Agric. Food Chem..

[3] D'Mello SA, Finlay GJ, Baguley BC, Askarian-Amiri ME (2016). Signaling pathways in melanogenesis. Int. J. Mol. Sci..

[4] Agar N, Young AR (2005). Melanogenesis: a photoprotective response to DNA damage. Mutat. Res..

[5] Maresca V, Flori E, Bellei B, Aspite N, Kovacs D, Picardo M (2010). MC1R stimulation by alpha-MSH induces catalase and promotes its re-distribution to the cell periphery and dendrites. Pigment Cell Melanoma Res..

[6] Galibert MD, Carreira S, Goding CR (2001). The Usf-1 transcription factor is a novel target for the stress-responsive p38 kinase and mediates UV-induced tyrosinase expression. EMBO J..

[7] Chung YC, Ko JH, Kang HK, Kim S, Kang CI, Lee JN (2018). Antimelanogenic effects of *Polygonum tinctorium* flower extract from traditional Jeju fermentation via upregulation of extracellular signal-regulated kinase and protein kinase B activation. Int. J. Mol. Sci..

[8] Ando H, Kondoh H, Ichihashi M, Hearing VJ (2007). Approaches to identify inhibitors of melanin biosynthesis via the quality control of tyrosinase. J. Invest. Dermatol..

[9] Del Marmol V, Ito S, Jackson I, Vachtenheim J, Berr P, Ghanem G (1993). TRP-1 expression correlates with eumelanogenesis in human pigment cells in culture. FEBS Lett..

[10] Lee SE, Hwang HJ, Ha JS, Jeong HS, Kim JH (2003). Screening of medicinal plant extracts for antioxidant activity. Life Sci..

[11] Yoon MY (2013). A study on anti-oxidant activity and anti-inflammatory action of sea buckthorn seed extract. KSBB J..

[12] Desmedt B, Rogiers V, Courselle P, De Beer JO, De Paepe K, Deconinck E (2013). Development and validation of a fast chromatographic method for screening and quantification of legal and illegal skin whitening agents. J. Pharm. Biomed. Anal..

[13] Zolghadri S, Beygi M, Mohammad TF, Alijanianzadeh M, Pillaiyar T, Garcia-Molina P (2023). Targeting tyrosinase in hyperpigmentation: current status, limitations and future promises. Biochem. Pharmacol..

[14] Ando S, Ando O, Suemoto Y, Mishima Y (1993). Tyrosinase gene transcription and its control by melanogenic inhibitors. J. Invest. Dermatol..

[15] Butler MS (2004). The role of natural product chemistry in drug discovery. J. Nat. Prod..

[16] Patel S, Rauf A, Khan H, Khalid S, Mubarak MS (2017). Potential health benefits of natural products derived from truffles: a review. Trends Food Sci. Technol..

[17] Lee H, Nam K, Zahra Z, Farooqi MQU (2020). Potentials of truffles in nutritional and medicinal applications: a review. Fungal Biol. Biotechnol..

[18] Park H, Gwon JH, Lee JC, Eom AH (2021). Report on a new truffle species, *Tuber koreanum* sp. nov., from Korea. Mycobiology.

[19] Park H, Gwon JH, Lee JC, Kim HS, Seo GS, Eom AH (2021). Morphological and phylogenetic characteristics of *Tuber himalayense* collected from rhizosphere of *Quercus dentata* in Korea. Kor. J. Mycol..

[20] Kim M, Hong H, Kim JH, Kim SY, Kim C (2021). Anti-inflammatory activity of indigenous *Tuber himalayense* in Korea. J. Mushrooms.

[21] Wang M, Carver JJ, Phelan VV, Sanchez LM, Garg N, Peng Y (2016). Sharing and community curation of mass spectrometry data with Global Natural Products Social Molecular Networking. Nat. Biotechnol..

[22] Videira IF, Moura DF, Magina S (2013). Mechanisms regulating melanogenesis. An. Bras. Dermatol..

[23] Maeda K, Fukuda M (1996). Arbutin: mechanism of its depigmenting action in human melanocyte culture. J. Pharmacol. Exp. Ther..

[24] Lee CJ, Park SK, Kang JY, Kim JM, Yoo SK, Han HJ (2019). Melanogenesis regulatory activity of the ethyl acetate fraction from *Arctium lappa* L. leaf on α-MSH-induced B16/F10 melanoma cells. Ind. Crops Prod..

[25] Tomita Y, Seiji M (1977). Inactivation mechanism of tyrosinase in mouse melanoma. J. Dermatol..

[26] Ngeow KC, Friedrichsen HJ, Li L, Zeng Z, Andrews S, Volpon L (2018). BRAF/MAPK and GSK3 signaling converges to control MITF nuclear export. Proc. Natl. Acad. Sci. USA.

[27] Mansky KC, Sankar U, Han J, Ostrowski MC (2002). Microphthalmia transcription factor is a target of the p38 MAPK pathway in response to receptor activator of NF-kappa B ligand signaling. J. Biol. Chem..

[28] Tachibana M (2000). MITF: a stream flowing for pigment cells. Pigment Cell Res..

[29] Yajima I, Kumasaka MY, Thang ND, Goto Y, Takeda K, Iida M (2011). Molecular network associated with MITF in skin melanoma development and progression. J. Skin Cancer.

[30] Kim HM, Moon MY, Hyun CG (2023). Citrulluside T, isolated from the *Citrullus lanatus* stem, inhibits melanogenesis in α-MSHinduced mouse B16F10 cells. Cosmetics.

[31] Fitzpatrick TB, Becker  SW, Lerner AB, Montgomery H (1950). Tyrosinase in human skin: demonstration of its presence and of its role in human melanin formation. Science.

[32] Kobayashi T, Urabe K, Winder A, Jiménez-Cervantes C, Imokawa G, Brewington T (1994). Tyrosinase related protein 1 (TRP1) functions as a DHICA oxidase in melanin biosynthesis. EMBO J..

[33] Ortonne JP, Ballotti R (2000). Melanocyte biology and melanogenesis: what's new?. J. Dermatolog Treat..

[34] Couteau C, Coiffard L (2016). Overview of skin whitening agents: drugs and cosmetic products. Cosmetics.

[35] Burger P, Landreau A, Azoulay S, Michel T, Fernandez X (2016). Skin whitening cosmetics: feedback and challenges in the development of natural skin lighteners. Cosmetics.

[36] Smit N, Vicanova J, Stan P (2009). The hunt for natural skin whitening agents. Int. J. Mol. Sci..

[37] Kim MY, Lee HE, Im M, Lee Y, Kim CD, Lee JH (2014). Effect of adenosine on melanogenesis in B16 cells and zebrafish. Ann. Dermatol..

